# Prone positioning combined with high-flow nasal cannula in severe noninfectious ARDS

**DOI:** 10.1186/s13054-020-2821-y

**Published:** 2020-03-23

**Authors:** Orlando R. Pérez-Nieto, Manuel A. Guerrero-Gutiérrez, Ernesto Deloya-Tomas, Silvio A. Ñamendys-Silva

**Affiliations:** 1 General Hospital of San Juan del Rio and Santo Tomas Hospital. Intensive care unit, Queretaro, Mexico; 2 Department of Critical Care Medicine, Instituto Nacional de Cancerologia, Mexico City, Mexico; 3 Department of Critical Care Medicine, Instituto Nacional de Ciencias Medicas y Nutricion Salvador Zubiran, Mexico City, Mexico; 4 Department of Critical Care Medicine, Hospital Medica Sur, 14050 Mexico City, Mexico

Dear editor,

We have read with exceptional interest the manuscript of Lin Ding et al. because the use of noninvasive ventilation (NIV) and high flow-nasal cannula (HFNC) combined with the prone position (PP) could avoid intubation of patients with acute respiratory distress syndrome (ARDS) [1].

The prone position is associated with a decrease in mortality in patients with ARDS, as demonstrated by Guerin in 2013 and the Formal Guide to the treatment of ARDS recommended the use of the PP for at least 16 h a day when P/F < 150 (moderate-severe ARDS) [2]. A study with a sample of 20 healthy patients shows an increase in lung volume at the end of expiration (LVEE) and a decrease in respiratory rate using HFNC demonstrated by electrical impedance tomography and that a PP resulted a more homogeneous distribution of the LVEE than the supine position [3].

The etiology of ARDS reported by Ding and colleagues [1] includes infectious pathologies in all cases, demonstrating that an early PP strategy with HFNC or NIV is safe and effective in patients with moderate ARDS and with SpO_2_ > 95% in which intubation could be avoided.

The mortality of ARDS associated with infectious etiology (pneumonia, influenza, and sepsis) is considerably higher (35.1% for pulmonary infection and 28.1% for sepsis) than that reported for noninfectious causes such as pneumonitis (6.4%) and trauma (2.5%) [4]; therefore, an early PP strategy combined with HFNC could theoretically be effective in these cases, even with P/F < 100.

We present a multicenter retrospective series of 6 cases of patients with severe ARDS with a noninfectious etiology compiled in 2017 and 2018 in hospitals of the 2nd level of care to which the PP was applied with HFNC or NIV. The PP was applied for 2–3 h every 12 h for 2 days, and in 3 cases, it was possible to avoid intubation; the causes were thoracic trauma with pulmonary contusions, lupus pneumonitis, bone marrow transplantation, and atelectasis of unknown cause (Table 1) (Fig. 1).

**Table 1:** Clinical characteristics and outcomes of patients

Case no.	Gender	Age (years)	Cause of ARDS	Ventilatory support	BaselinePaO2/FiO2 (P/F) (mmHg)	Baseline S/F	P/F after prone position with HFNC o NIV	S/F after prone position with HFNC or NIV	Beginning of prone position and HFNC or NIV	Intubation	Outcome
1	Male	33	Closed thorax trauma	HFNC	–	195	–	213	< 24 h	No	Survive
2	Female	19	Lupus pneumonitis	HFNC	91	133	150	165	< 24 h	No	Survive
3	Male	56	Open thorax trauma	HFNC	80	98	101	128	48 h	Yes	Survive
4	Female	36	Bone marrow transplant	NIV	67	87	96	155	72 h	Yes	Death
5	Male	45	Bilateral atelectasis	NIV	89	150	–	250	72 h	No	Survive
6	Male	24	Near drowning	HFNC	75	93	131	188	< 24 h	No	Survive

*ARDS* acute respiratory distress syndrome, *S/F* oxygen saturation ratio by pulse oximetry between inspired oxygen fraction, *HFNC* high-flow nasal cannula, *NIV* noninvasive ventilation

**Fig. 1:**
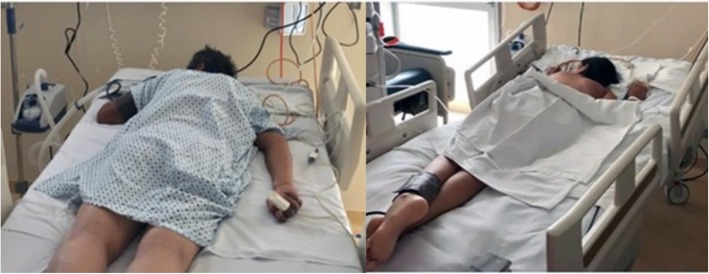
Patients with ARDS with high flow oxygen cannula and prone position

The PP with HFNC or NIV seems to be a promising strategy to avoid intubation and its complications in patients with severe ARDS of noninfectious etiology, and a randomized controlled study is required to assess its safety and efficacy. The results of the OPTIPRONE study on the use of PP combined with HFNC in patients with ARDS with PaO_2_/FiO_2_ < 200 [5] are expected.

## Authors’ response

Lin Ding and Hangyong He

We appreciate the time the authors have taken to read and comment on our recent article published in critical care [6].

First of all, which etiology of acute respiratory distress syndrome (ARDS) should be the most appropriate group treated with prone position (PP) combined with high-flow nasal cannula (HFNC)? In our study, the majority of ARDS were caused by infectious disease. And we totally agree that PP combined with HFNC should be tried in noninfectious ARDS patients, which was reported in previous studies and case series [7–9]. However, as reported by Perez-Nieto et al., the use of prone positioning of the patients with complex chest traumas and post status of thoracic surgery is sparse and relatively controversial [8]. Thus, its safety should be evaluated in these noninfectious ARDS population with special protocol.

Another question is whether it is safe and effective enough for patients with noninfectious ARDS with PaO_2_/FiO_2_ < 100. PP is a respiratory support technique but not for treating the causative disease which induced ARDS. Thus, as reported in the case series of Perez-Nieto et al., some group of noninfectious ARDS caused by autoimmune diseases (such as lupus pneumonitis) may need a longer duration of disease resolving than infectious disease and may present with deterioration even under PP and HFNC therapy. And patients with PaO_2_/FiO_2_ < 100 may face a delayed intubation and worse outcome. Therefore, the safety and efficacy of PP combined with HFNC in noninfectious diseases which cause severe ARDS in patients with a PaO_2_/FiO_2_ < 100 also need evaluation.

## Data Availability

Not applicable.
